# Non-Surgical Periodontal Treatment Outcomes in Patients with HIV Under Antiretroviral Therapy: A Systematic Review

**DOI:** 10.3390/jcm15020651

**Published:** 2026-01-14

**Authors:** Thaleia Angelopoulou, Yiorgos A. Bobetsis

**Affiliations:** 1 School of Medicine, National and Kapodistrian University of Athens, 11527 Athens, Greece; thangelop@yahoo.gr; 2 Department of Periodontology, School of Dentistry, National and Kapodistrian University of Athens, 11527 Athens, Greece

**Keywords:** HIV, periodontitis, periodontal treatment, antiretroviral therapy, viral load, CD4+ lymphocytes, systematic review

## Abstract

**Background/Objectives**: This systematic review aimed to evaluate the clinical and immunological outcomes of non-surgical periodontal therapy (NSPT) in HIV-positive patients with periodontitis. **Methods**: Systematic search on four databases (PubMed, Scopus, Web of Science, Cochrane Library) and the gray literature was completed through December 2025. A comprehensive set of clinical parameters and immunological markers were assessed. Three studies met the inclusion criteria and were included in the final synthesis and qualitative analysis. Extracted outcomes included clinical periodontal parameters (PPD, CAL, BoP, PI, GBI, BI) and immunological markers (viral load, CD4+ lymphocyte count, CD4/CD8 ratio, salivary LF, salivary HST, GCF LF, GCF HST). **Results**: With a very low level of certainty, NSPT was generally associated with significant improvements in clinical periodontal parameters compared to before treatment measurements and HIV-negative individuals. Improvements in immunological status were also reported. Heterogeneity of study designs and reporting standards limited this study’s quantitative analysis. **Conclusions**: NSPT demonstrates beneficial clinical and immunological outcomes in people living with HIV. However, the very low level of certainty in the available data limits confidence in changes in periodontal status and immune system reconstitution following NSPT in this population; therefore, the findings remain inconclusive and should be interpreted with caution.

## 1. Introduction

HIV initially drew global attention in the early 1980s, when the first cases of AIDS were reported. In recent decades, immense scientific progress has been made in the development of antiretroviral therapy, leading to a substantial reduction in HIV-related mortality, significantly improving the quality of life of affected individuals [1,2,3]. The number of new infections continues to rise each year, and around 40 million people are estimated to be currently living with HIV, while several projections expect this number to reach 44.4 million by 2039 [4]. Although a large proportion of these individuals are receiving antiretroviral treatment, around one-quarter of people living with HIV (PLWH) worldwide remain undiagnosed or lack access to life-saving therapies [5]. Given its systemic nature and impact on immune function, HIV infection is also associated with a broad spectrum of oral manifestations, including various oral lesions, opportunistic infections, and neoplasms, which often reflect the degree of immunosuppression and serve as the first clinical signs of the condition [6,7,8]. Necrotizing periodontal diseases and periodontitis have also been strongly associated with HIV infection [9].

Periodontitis is a chronic inflammatory disease characterized by the progressive destruction of tooth-supporting structures [10]. Non-surgical periodontal therapy (NSPT), including scaling and root planing (SRP), is the gold standard for the treatment of periodontitis, as subgingival instrumentation has been consistently shown to reduce periodontal inflammation and achieve effective infection control in patients with periodontitis, leading to a significant improvement in both periodontal and radiographic parameters [11,12,13,14]. In addition to its local clinical benefits, NSPT is also associated with significant effects on systemic inflammation, inducing both transient and long-term changes in circulating inflammatory markers, endothelial function, and modulation of platelet-related responses [15,16,17,18,19,20,21].

In PLWH, periodontitis has been shown to present with increased prevalence, as the virus-induced immunosuppression leads to an impaired regulation of inflammatory response, resulting in accelerated clinical attachment level and bone loss [22,23,24,25,26,27,28,29]. Since the introduction of highly active antiretroviral therapy (HAART) [30], the prevalence of HIV-associated oral lesions has changed substantially [31,32,33,34,35]. Periodontitis, however, remains highly prevalent among PLWH, regardless of antiretroviral treatment status [36,37,38,39]. Even though HAART appears to contribute to the improvement in some periodontal parameters [36,40,41], clinical evidence regarding the magnitude and consistency of this effect remains limited and conflicting [42,43,44,45,46,47,48]. This inconsistency can be attributed to the combined influence of methodological heterogeneity across studies as well as the considerable variability in immune reconstitution achieved through HAART among PLWH, since immune recovery is often partial [49,50]. HAART alone may not be sufficient to hinder periodontal disease progression, thus making periodontal treatment essential for managing periodontitis in this population.

Several studies have reported the beneficial impact of NSPT on clinical periodontal parameters in this population, while others have demonstrated a positive influence on immune function, suggesting that improving periodontal health and reducing local inflammation may contribute to systemic immune reconstitution. However, no systematic review has yet addressed this topic. Therefore, the aim of this systematic review was to assess the clinical and immunological outcomes of NSPT in HIV-positive patients with periodontitis receiving antiretroviral therapy, as well as to evaluate differences in outcomes compared with HIV-negative patients.

## 2. Materials and Methods

### 2.1. Protocol and Registration

This systematic review was conducted in accordance with the Preferred Reporting Items for Systematic Reviews and Meta-Analyses (PRISMA) 2020 guidelines (File S1) [51]. The study protocol was registered in the International Prospective Register of Systematic Reviews (PROSPERO) database under registration number CRD420251155734.

### 2.2. Study Design

A systematic review was conducted to answer the following research question: “Does NSPT lead to changes in clinical and immunological outcomes in HIV-positive patients with periodontitis receiving antiretroviral therapy, and are there differences in these outcomes compared with HIV-negative patients?”.

### 2.3. Eligibility Criteria

#### 2.3.1. Inclusion Criteria

The inclusion criteria for this systematic review were based on the PICOS framework (Population, Intervention, Comparison, Outcomes, and Study design). Prospective clinical studies, randomized controlled trials (RCTs), case–control studies, cross-sectional studies, and cohort studies (S) assessing the effect of NSPT (I) on clinical and immunological parameters (O) in HIV-positive patients receiving antiretroviral therapy, who were also diagnosed with periodontitis (P), with comparisons based on changes from their pre-intervention baseline values and on post-treatment outcomes reported in HIV-negative patients with periodontitis who also received periodontal treatment (C), were included in this systematic review. Studies were additionally required to have clearly defined intervention protocols, specifying NSPT as comprising supra-gingival biofilm control, subgingival scaling, and root planing using hand instruments and/or ultrasonic scalers, combined with the provision of oral hygiene instruction. Eligible studies also fulfilled the following inclusion criteria: studies that were conducted in adult populations (≥18 years old); studies that included patients with a minimum of 12 natural teeth; studies in which periodontitis was diagnosed based on clinical parameters; and studies with a minimum follow-up period of 3 months after treatment. Only peer-reviewed articles written or translated into the English language were considered.

#### 2.3.2. Exclusion Criteria

The exclusion criteria for this systematic review were as follows: studies with designs different from those specified in the inclusion criteria (systematic reviews, reports, letters, editorials, commentaries, and case reports); studies in which patients received surgical periodontal therapy; studies where the participants had received antibiotic treatment within the preceding six months (except for drugs prescribed as prophylaxis for opportunistic infections in individuals with HIV, which could be allowed and maintained during the protocol); studies where patients were diagnosed with additional systematic conditions or comorbidities known to influence the progression or severity of periodontitis, such as uncontrolled diabetes mellitus, cardiovascular diseases, immunosuppressive disorders other than HIV infection, or any other autoimmune diseases; studies where the patients were diagnosed with necrotizing periodontitis, frequently associated with HIV infection, or any other form of periodontal disease; studies involving smoking patients; studies lacking clear intervention protocols; and in vitro studies and animal studies.

### 2.4. Information Sources and Search Strategy

The search was completed through 22 December 2025. Detailed individual search strategies for each of the following databases were developed: PubMed, Web of Science, Scopus, and Cochrane Library. A partial gray literature search was performed using Base, ProQuest, ResearchGate, and Google Scholar (Table S1). Studies were collected, and duplicate hits were removed using reference manager software (Mendeley Refence Manager Version 2.135.0). Duplicates not identified by the software were manually removed.

### 2.5. Study Selection

**Figure 1:**
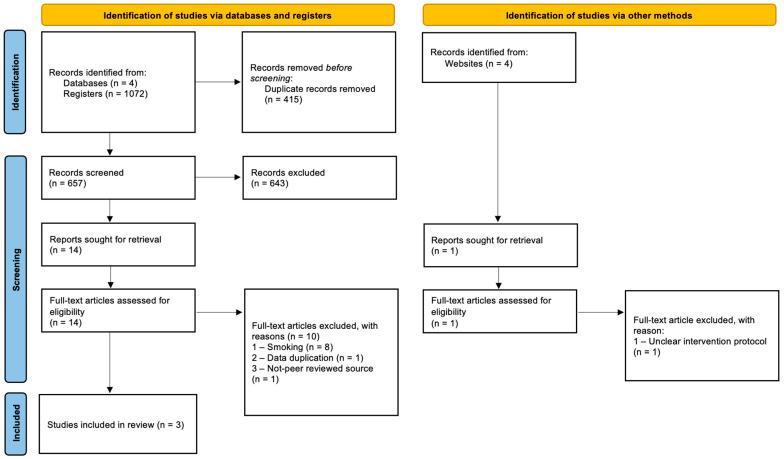
Flow diagram of literature search adapted from PRISMA.

Study selection was managed in two separate phases. In the first phase, the two authors independently reviewed the titles and abstracts of articles that appeared to meet the eligibility criteria, and in the second phase, they independently read the full text of the articles selected in phase 1 to assess their eligibility. Discrepancies were resolved through discussion until consensus was reached between the two reviewers. The reference lists of the included studies were manually assessed by one author (T.A.) to identify any studies that may have been unintentionally omitted. The entire selection process was documented in accordance with the PRISMA guidelines and is illustrated in the PRISMA flow diagram (Figure 1).

### 2.6. Data Collection Process and Data Items

The two reviewers independently extracted data from each included study using a standardized data extraction form developed in a Microsoft Excel spreadsheet. The following information was collected: bibliographic details (authors, language, country, year of publication and journal name), study design, sample size, and sample characteristics relevant to the PICO framework, including population demographics, periodontitis definition and HIV profile, number of teeth per participant, presence of comorbidities or other systematic conditions potentially affecting treatment outcomes, smoking status, and antibiotic usage. Additional data items included the clinical criteria used for periodontitis diagnosis, periodontal intervention protocol details, characteristics of the comparator group, clinical and immunological outcomes, and follow-up duration. Disagreements regarding the data extraction were resolved by discussion, and final study selection was decided after there was mutual agreement between the reviewers. Cohen’s kappa coefficient was used to assess the agreement value between the two evaluators and inter-rater reliability during the screening phase [52].

### 2.7. Risk of Bias Within Studies

The risk of bias in each included study was assessed independently by the two reviewers. The Risk of Bias in Non-randomized Studies of Interventions (ROBINS-I) tool [53] was used to assess the methodological quality of non-randomized clinical trials based on seven bias domains, leading to an overall judgment of “low”, “moderate”, “serious”, or “critical” risk of bias for each study.

### 2.8. Summary Measures

The primary outcomes of this systematic review were the change in probing pocket depth (PPD, mm) following NSPT. Clinical attachment level (CAL, mm) measured with a calibrated periodontal probe, bleeding on probing (BoP, % of sites), plaque index (PI, % of sites), gingival bleeding index (GBI, % of sites), and bleeding index (BI, % of sites) were considered secondary outcomes. The secondary outcomes also included changes in immunological parameters, including viral load (copies/mL plasma), CD4+ lymphocyte count (cells/μL blood), CD4/CD8 ratio, salivary lactoferrin (LF, μg/mL), salivary histatin (HST, pg/mL), and gingival crevicular fluid (GCF) concentrations of LF (pg/mL) and HST (pg/mL) following NSPT.

### 2.9. Certainty of the Evidence

The certainty of the evidence for the outcomes included in this systematic review was assessed using the GRADE system, and a Summary of Findings (SoF) table was generated by the GRADE-pro website (https://www.gradepro.org, accessed on 10 October 2025). The certainty of the evidence for each outcome was classified as high, moderate, low, or very low based on the GRADE criteria (risk of bias, imprecision, inconsistency, indirectness, imprecision, publication bias, large effect, plausible confounding, and dose–response gradient).

## 3. Results

### 3.1. Study Selection

A total of 1072 references were retrieved from the four electronic bases (PubMed, Scopus, Web of Science, Cochrane Library), and one was manually added from searching across the gray literature. After the removal of duplicate studies and evaluation of abstracts, the first screening phase led to 15 studies being eligible for full-text evaluation. In the second phase, 11 studies were excluded from the final selection because of participants’ smoking status (n = 8), data duplication (n = 1), studies being published in a non-peer-reviewed source (n = 1), and having an unclear intervention protocol (n = 1) (Table S2). Three studies were eligible for qualitive synthesis. In the selection process, complete agreement between reviewers was achieved (Kappa > 0.90). No additional references were retrieved from the reference lists of included studies.

### 3.2. Study Characteristics

Included studies were published between 2006 and 2023, and they were all published in English. The first study, by Jordan et al. [54], was a prospective, non-randomized, parallel-group, controlled clinical trial conducted in Germany, and it included 22 participants. Jordan et al. defined periodontitis cases as those having at least four teeth presenting with PPD ≥ 4 mm. All participants received oral hygiene instruction (OHI), NSPT, and supportive periodontal treatment (SPT). All HIV-seropositive participants were receiving highly active antiretroviral therapy (HAART). Patients who had used antibiotics within the preceding 6 months were excluded, while information regarding participants’ smoking status was not provided. Data were collected from both the test group and the control group pre- and post-intervention. The study included a follow-up period of 15 months. The second included study was a prospective, split-mouth, non-randomized, controlled pilot trial conducted by Salgado et al. [55] in Brazil, including 12 participants diagnosed with mild periodontitis, classified according to the Eke et al. classification [56]. In this trial, the intervention group received OHI and NSPT, followed by the application of photodynamic therapy (PDT) with malachite green (MG) at 0.01%, whereas the control group received the same treatment without PDT. All HIV-seropositive participants were receiving HAART for more than 3 years. The number of affected teeth or sites of participants included in this study was not specified. Only data from the comparison group (control group) pre- and post-intervention were collected, as the intervention (test group) did not meet the PICOS criteria of this systematic review. The study included a maximum follow-up period of 3 months. The last included study, by Nobre et al. [57,58], was a prospective, non-randomized, parallel-group, controlled clinical trial conducted in Brazil. Its results were reported in two separate publications [57,58], each analyzing different outcomes and reporting different sample sizes; however, the number of participants infected with HIV remained the same across both reports. The study included a total sample size of 47 participants who met the diagnostic criteria for chronic periodontitis, defined by a combination of PPD and CAL according to the classification by Armitage et al. [59], later re-evaluated according to the 2018 World Workshop classification [60]. All participants received OHI, NSPT, and SPT. All HIV-seropositive participants were receiving HAART for more than 3 years. Patients who had used antibiotics within the preceding 6 months were excluded. Data were collected from both the test group and the control group pre- and post-intervention. The study included a maximum follow-up period of 3 months. The main characteristics of the included studies are summarized in Table 1. Heterogeneity of study designs and reporting standards limited this study’s quantitative analysis.

### 3.3. Risk of Bias Within Studies

**Figure 2:**
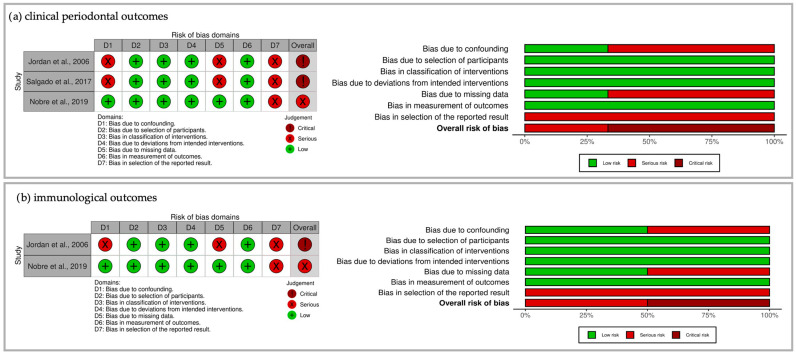
(**a**) Risk of bias assessment of the included non-randomized clinical trials evaluated using the Cochrane ROBINS-I V2 tool and visualized with the robvis tool regarding the clinical periodontal outcomes of each study [54,55,57]. Figures on the left: Risk of bias summary: review authors’ judgments about each risk of bias domain for each included study regarding the clinical outcomes. Figures on the right: Risk of bias graph: review authors’ judgments about each risk of bias domain presented as percentages across all included studies regarding the clinical periodontal outcomes; (**b**) risk of bias assessment of the included non-randomized clinical trials evaluated using the Cochrane ROBINS-I V2 tool and visualized with the robvis tool regarding the immunological outcomes of each study [54,57]. Figures on the left: Risk of bias summary: review authors’ judgments about each risk of bias domain for each included study regarding the immunological outcomes. Figures on the right: Risk of bias graph: review authors’ judgments about each risk of bias domain presented as percentages across all included studies regarding the immunological outcomes.

The three non-randomized clinical trials were assessed using the Cochrane ROBINS-I V2 tool [53]. The risk of bias for clinical and immunological outcomes was assessed separately. The results were summarized and visualized using the robvis tool [61] in Figure 2. Regarding the clinical periodontal outcomes, two studies [54,55] were assessed as having critical risk of bias due to confounding, missing data, and selection of reported results and one [57] was considered to have serious risk of bias, also due to bias in the selection of the reported results. The overall risk of bias of the two studies that assessed immunological outcomes was judged as critical [54] and serious [57] for the same reasons.

### 3.4. Synthesis of Results and Assessment of the Certainty of Evidence

#### 3.4.1. Changes in Clinical Periodontal Parameters

In the study of Jordan et al. [54], including 22 participants, mean PPD was significantly reduced in both the test (from 4.2 ± 0.3 mm at baseline to 2.6 ± 0.4 mm post-treatment) and the control group (from 4.3 ± 0.3 mm at baseline to 2.6 ± 0.4 mm postoperatively) over a 15-month observation period. Intragroup PPD reduction was highly significant (*p* = 0.0005); however, this was not the case for intergroup comparisons. BoP was improved in both groups (decreasing from >50% bleeding sites at baseline to approximately 20% post-treatment). The level of certainty in the evidence for both outcomes of this study was rated as very low (Table S3). Salgado et al. [55], in a study comprising 12 participants, found that PPD was reduced one week after NSPT (from 4.5 ± 0.5 mm at baseline to 3.9 ± 0.5 mm). After 3 months, PI reduction was not statistically significant, whereas a significant reduction in GBI was observed. The level of certainty in the evidence for the outcomes of this study was rated as very low (Table S3). Nobre et al. [57], reporting on a sample of 22 HIV-positive and 20 HIV-negative participants, demonstrated improvement in GBI, PPD, and CAL following NSPT, although these clinical changes were only presented descriptively. It was in a subsequent publication [58], including the original 22 HIV-positive participants and an updated count of 25 HIV-negative participants, where quantitative data were provided, thus allowing for a more detailed assessment of the clinical outcomes. NSPT improved the clinical parameters of periodontal disease in both groups. PPD, CAL, and BI significantly decreased across the three time points (*p* < 0.01) in both groups, with a small but statistically significant intragroup difference for PPD (*p* = 0.04); no intragroup differences were observed for CAL (*p* = 0.11) or BI (*p* = 0.31). In the HIV-positive group (test group), PPD decreased from 2.8 ± 0.6 mm at baseline to 2.2 ± 0.6 mm at 30 days and 2.0 ± 0.6 mm at 90 days following NSPT. CAL improved from 3.1 ± 0.7 mm at baseline to 2.7 ± 0.7 mm at 30 days and 2.6 ± 1.0 mm at 90 days. BI was reduced from 35.4% at baseline to 10.3% at 30 days and 12.7% at 90 days. In the HIV-negative group (control group), PPD decreased from 2.8 ± 0.6 mm at baseline to 2.6 ± 0.6 mm at 30 days and 2.4 ± 0.5 mm at 90 days after NSPT. CAL improved from 3.3 ± 0.9 mm at baseline to 3.1 ± 0.8 mm at 30 days and 3.0 ± 0.9 mm at 90 days. BI decreased from 29.0% at baseline to 10.7% at 30 days and 9.1% at 90 days following NSPT. The level of certainty in the evidence for the outcomes of this study was rated as very low (Table S3).

#### 3.4.2. Changes in Immunological Parameters

Jordan et al. [54] found a considerable degree of stability in the immunologic markers associated with HIV infection over their 15-month-long study. CD4+ lymphocyte count and the CD4/CD8 ratio showed relative stability, while a small reduction in viral load was observed. In the first publication of Nobre et al.’s study [57], the salivary levels of IL-6 and IL-8 showed a tendency to increase at the 30-day time point, followed by a tendency to decrease at 90 days after NSPT in both groups. TNF-α was present at very low concentrations in saliva in the test group, whereas it was undetectable in the control group. Despite these low detection levels, an increase was observed at 30 days, followed by a decrease at 90 days after NSPT. No significant differences were found in the concentrations of IL-6 (*p* = 0.68) or TNF-α (*p* = 0.70) over time, whereas a significant decrease in salivary IL-8 was observed in both groups (*p* = 0.05). In the same publication, a statistically significant increase in the CD4+ T-lymphocyte count of HIV-positive patients was reported (*p* = 0.0120) (from 104.7 ± 57.3 cells/mm^3^ at baseline to 165.7 ± 110.4 cells/mm^3^ at 30 days and 195.6 ± 155.2 cells/mm^3^ at 90 days following NSPT). A reduction in viral load was also observed over time (from 1,444,892.2 ± 423,174.5 copies/mL at baseline to 19,547.4 ± 66,181.4 copies/mL at 30 days and 28,380.8 ± 103,229.3 copies/mL at 90 days); however, this change was not statistically significant (*p* = 0.2984). In the subsequent publication of Nobre et al. [58], conducted with a slightly larger sample of HIV-negative participants, a tendency toward reduction in salivary lactoferrin (LF) was reported in both groups. In the HIV-positive group (test group), LF levels decreased from 4.1 μg/mL at baseline to 2.6 μg/mL at 30 days and 2.2 μg/mL at 90 days following NSPT. In the HIV-negative group, LF levels decreased from 3.0 μg/mL at baseline to 2.7 μg/mL at 30 days and 1.8 μg/mL at 90 days. However, this reduction did not reach statistical significance (*p* = 0.51). In contrast, salivary and GCF levels of HST, as well as GCF levels of LF, were significantly higher in HIV-positive patients compared with HIV-negative individuals. More specifically, in the test group of patients with HIV, the salivary HST concentrations changed from 62.6 × 10^2^ pg/mL at baseline to 59.8 × 10^2^ pg/mL at 30 days and 62.3 × 10^2^ pg/mL at 90 days after NSPT. In the control group of patients who were not infected with HIV, the salivary HST concentrations were considerably lower, measured at 22.4 × 10^2^ pg/mL at baseline, 20.1 × 10^2^ pg/mL at 30 days, and 20.1 × 10^2^ pg/mL at 90 days (*p* < 0.01). For GCF LF, the HIV-positive group showed values of 8.8 × 10^2^ pg/mL at baseline, 10.3 × 10^2^ pg/mL at 30 days, and 8.8 × 10^2^ pg/mL at 90 days following NSPT, whereas the HIV-negative group exhibited consistently lower values measured at 6.7 × 10^2^ pg/mL at baseline, 5.4 × 10^2^ pg/mL at 30 days, and 5.7 × 10^2^ pg/mL at 90 days (*p* = 0.01). Regarding GCF HST, the HIV-positive group exhibited an increase from 97.8 × 10^2^ pg/mL at baseline to 135.4 × 10^2^ pg/mL at 30 days, followed by a return to 110.0 × 10^2^ pg/mL at 90 days after NSPT. The HIV-negative group recorded 25.8 × 10^2^ pg/mL at baseline, 39.5 × 10^2^ pg/mL at 30 days, and 22.5 × 10^2^ pg/mL at 90 days (*p* = 0.01). The level of certainty in the evidence for immunological outcomes was rated as very low (Table S3).

## 4. Discussion

### 4.1. Summary of Evidence

This systematic review evaluated the effect of NSPT on clinical periodontal and immunological parameters in HIV-positive patients with periodontitis. The values of standard markers were compared to both the pre-intervention measurements of HIV-positive participants and those of HIV-negative patients. Three studies were critically appraised. All of them assessed clinical periodontal indices, and two also analyzed immune markers. NSPT led to consistent short-term improvements in periodontal parameters among HIV-positive patients, including reductions in PPD, CAL, and bleeding indices, with clinical responses being comparable to those observed in HIV-negative individuals. However, given the minimal intergroup differences, the findings indicate that NSPT improves periodontal health regardless of HIV status. Immunological outcomes were overall inconsistent across studies. CD4+ lymphocyte counts remained stable or increased following NSPT, and viral load declined. Levels of cytokines and antimicrobial proteins exhibited heterogenous patterns, though HIV-positive individuals consistently showed higher HST and GCF lactoferrin levels than HIV-negative participants. The findings of the present systematic review suggest that NSPT has a beneficial effect on both periodontal and immunological profiles of PLWH, although confidence in this evidence remains very low.

These results are in agreement with data reported in the existing literature. Noro Filho et al.’s study [62], which could not be included in the present systematic review as its sample included smokers, provided evidence of systemic immune enhancement after NSPT in individuals infected with HIV receiving antiretroviral therapy. A statistically significant increase in CD4+ lymphocyte counts was detected 3 and 6 months post-treatment, whereas viral load, without statistical significance, was reduced, becoming undetectable in all participants by 6 months. Moreover, a significant positive correlation was found between PPD and viral load reduction, as well as the increase in CD4+ lymphocyte counts, confirming the link between periodontal improvement and systemic immune recovery. In the study of Valentine et al. [63], which also could not be included in the present review because its population and study design did not meet our eligibility criteria, the researchers highlighted the significant association between high plasma viral load with severe periodontal disease and also reported a significant reduction in IL-6 levels and viral load in HIV-positive patients following NSPT. The increase in CD4+ lymphocyte counts reached statistical significance 24 months post-treatment, suggesting a delayed systemic effect of periodontal inflammation resolution combined with the well-established ongoing benefits of antiretroviral therapy. However, Shintani et al. [64] found no relationship between periodontal indices and CD4+ cell counts, suggesting that local periodontal inflammation and thus its resolution may not be directly reflected in systemic immune status. To the best of our knowledge, to date, there is no other systematic review in the literature evaluating clinical periodontal and immunological outcomes of non-surgical periodontal therapy (NSPT) in HIV-positive patients with periodontitis.

Periodontitis is a chronic inflammatory disease of the periodontal apparatus which is initiated and modulated by a complex, dynamic relationship between bacterial biofilm accumulation and the host’s immune response [10]. Given that immune function plays a vital role in the pathogenesis of periodontal disease, individuals with compromised immune responses, as in the case of HIV infection, tend to exhibit a higher prevalence and greater severity of periodontal destruction [22,23,24,25,26,27,28,29]. Although antiretroviral therapy HAART has a well-documented beneficial effect on HIV-related oral lesions [31,32,33,34,35], periodontitis still remains highly prevalent among PLWH [36,37,38,39]; however, clinical evidence of this effect remains scarce and conflicting [42,43,44,45,46,47,48]. This inconsistency can be attributed to both methodological heterogeneity among studies and variability in immune reconstitution in PLWH [49,50], affecting not only the prevalence and severity of periodontitis but also the response to periodontal therapy. Evidence suggests that PLWH who develop resistance to HAART exhibit lower CD4+ lymphocyte counts and higher viral loads, indicating incomplete immune recovery despite ongoing therapy. Their altered immune response negatively affects periodontal tissues and therefore may also affect response to NSPT [65,66]. Other possible explanations for the absence of highly significant results in this review may include methodological limitations related to study design, high risk of bias, and very low certainty of evidence.

### 4.2. Limitations and Strengths

Despite the high biological plausibility supporting the link between host–immune modulation and periodontal inflammation resolution, the interpretability and generalizability of the findings of this review are substantially limited and weakened by the characteristics of the available evidence. After conducting a systematic search of scientific databases, only three studies were included in the present review, resulting in a small sample size. The studies also presented with significant methodological heterogeneity regarding study design, participant characteristics, intervention protocols, outcome definitions, and follow-up duration. It is important to note that all the studies included in this review were also assessed as having a serious to critical risk of bias and very low certainty of evidence, further limiting the reliability of our conclusions. Therefore, the findings should be interpreted with caution and viewed primarily as descriptive rather than confirmatory.

In addition, none of the three studies included were originally designed to evaluate the full scope of periodontal and immunological responses to NSPT in HIV-positive patients compared with HIV-negative controls. As a result, the available data were not adequately powered or adjusted for intergroup comparisons, and in some cases, the outcomes had to be interpreted as within-group changes rather than between-group effects. This methodological limitation also weakens the strength of our conclusions.

In this systematic review, antiretroviral regimens varied across studies. In all three studies, the patients were receiving antiretroviral therapy, and in two of them, this therapy had been received for more than three years [54,57]. While Salgado et al. [55] did not specify the regimen used, Jordan et al. [54] reported that their patients received a combination of non-nucleoside reverse transcriptase inhibitors (NNRTIs) and protease inhibitors (PIs). Nobre et al. [57] stated that the most frequent combination included nucleoside analog reverse transcriptase inhibitors (NRTIs) and protease inhibitors (PIs), and the most common drugs in this combination were tenofovir (TDF), lamivudine (3TC), and atazanavir (ATV). The second most frequent combination used was protease inhibitors (PIs) combined with integrase inhibitors (INSTIs). However, discrepancies in the cART regimens among participants infected with HIV may have influenced the observed outcomes.

Moreover, various clinical thresholds were used to define periodontitis among participants. Hence, Jordan et al. [54] included patients with periodontitis, defined as the presence of at least four teeth with PPD ≥ 4 mm, Salgado et al. [55] included patients with mild periodontitis, classified according to the Eke et al. classification [56], and Nobre et al. [57] included individuals who met the diagnostic criteria for chronic periodontitis, defined by a combination of PPD and CAL according to the classification by Armitage et al. [59], later re-evaluated according to the 2018 World Workshop classification [60]. It is obvious that differences in the initial severity of periodontitis may have affected treatment outcomes and therefore the level of periodontal and systemic inflammation post-treatment. Hence, accurate comparisons among these studies and generalization of outcomes may be misleading.

Another important limitation of the included studies was the short follow-up duration, which ranged from 3 months to 15 months. Studies with longer follow-up periods are needed to better portray the potential delayed systemic effects of periodontal therapy in immunological parameters of HIV-positive patients.

Regarding the strengths of this systematic review, a comprehensive search was conducted covering four major scientific databases and the gray literature, with no language or time restrictions. All screening, data extraction, and risk of bias assessments were performed independently and in duplicate. Discrepancies were resolved through discussion until consensus was reached between the two reviewers. Additionally, standard Cochrane tools were used to assess risk of bias as well as the quality of the included studies. All included studies were analyzed in detail with respect to clinical, immunological, and methodological parameters, allowing for a thorough synthesis of the available evidence.

## 5. Conclusions

HIV-associated periodontal diseases are recognized as significant complications of HIV infection. As the life expectancy of PLWH continues to increase, the management of periodontal inflammation and the prevention of periodontitis are becoming important clinical concerns. Although several studies support that NSPT demonstrates a beneficial impact not only on clinical periodontal but also immunological outcomes in PLWH, the evidence supporting a causal association between NSPT and immune system reconstitution in this population remains very poor and inconclusive. Given the limited and methodologically inconsistent evidence available, well-designed clinical trials are needed to clarify the potential role of NSPT in improving periodontal status and promoting immune reconstitution in this population.

## Figures and Tables

**Table 1:** Summary of descriptive characteristics of included studies (n = 3).

Study/ Country	Study Design	Study Population, Intervention and Control Groups, n	Age (Years)	Periodontitis Definition and HIV Profile	NSPT Protocol	Clinical Periodontal and Immunological Outcomes	Follow-Up (Months)	Main Conclusion
Jordan et al., 2006 [54] Germany	Prospective non-randomized interventional parallel-group controlled clinical trial	22 participants: 11 HIV+ patients 11 HIV− patients	≤45 (Mean: 37.8)	≥4 teeth presenting PPD ≥ 4 mm HIV-1 seropositivity; exposure to HAART	OHI, SRP, SPT	PPD, GΒI, BoP, PI CD4+ lymphocyte count, CD/CD8 ratio, HIV viral load	15	Non-surgical periodontal therapy has been found to be effective in improving clinical periodontal parameters and enhancing the immunological profile of HIV-positive patients with periodontitis receiving antiretroviral therapy.
Salgado et al., 2017 [55] Brazil	Prospective split-mouth non-randomized interventional controlled pilot trial	12 HIV+ participants	48.6 ± 5.8	Mild Periodontitis (Eke et al., 2012, [56] classification); ≥2 contralateral sites presenting PPD ≥ 4 mm HIV-1 seropositivity; exposure to HAART ≥ 3 years	OHI, SRP (control group) OHI, SRP, PDT w/0.01% MG (intervention group)	PPD, BoP, GBI, PI Not Reported	0.25, 1, 3	Non-surgical periodontal therapy has been found to be effective in improving clinical periodontal parameters in HIV-positive patients with periodontitis receiving antiretroviral therapy.
Nobre et al., 2019 [57] Nobre et al., 2023 [58] Brazil	Prospective non-randomized interventional parallel-group controlled clinical trial	42 participants: 22 HIV+ patients 20 HIV− patients (Nobre et al., 2019 [57]) 47 participants: 22 HIV+ patients 25 HIV− patients (Nobre et al., 2023 [58])	46.2 ± 9.7 44.8 ± 5.8 (intervention group) 47.8 ± 12.8 (control group)	Chronic Periodontitis (Armitage et al., 1999, [59] classification); ≥2 sites presenting PD ≥ 5 mm and ≥2 teeth presenting CAL ≥ 5 mm, re-evaluated according to the 2018 World Workshop classification (Caton et al., 2018 [60]). HIIV-1 seropositivity; exposure to cART (NRTI, NNRTI, INSTI, PI) ≥ 3 years	OHI, SRP, SPT	PD, CAL, GBI (Nobre et al., 2019 [57]) PD, CAL, BI (Nobre et al., 2023 [58]) Salivary IL-6; Salivary IL-8; Salivary TNF-α, CD4+ lymphocyte count, HIV viral load (Nobre et al., 2019 [57]) Salivary LF; Salivary HST; GCF LF; GCF HST (Nobre et al., 2023 [58])	3	Non-surgical periodontal therapy has been found to be effective in improving clinical periodontal parameters and enhancing the immunological profile of HIV-positive patients with periodontitis receiving antiretroviral therapy.

HAART, highly active antiretroviral therapy; PPD, probing pocket depth; CAL, clinical attachment level; BoP, bleeding on probing; PI, plaque index; GBI, gingival bleeding index; ΒΙ, bleeding index; OHI, oral hygiene instruction; SRP, scaling and root planing; SPT, supportive periodontal therapy; PDT, photodynamic therapy; MG, malachite green; IL-6, interleukin 6; IL-8, interleukin 8; TNF-α, tumor necrosis factor alpha; GCF, gingival crevicular fluid; HST, histatin; LF, lactoferrin; cART, combination antiretroviral therapy; NRTIs, nucleoside reverse transcriptase inhibitors; NNRTIs, non-nucleoside reverse transcriptase inhibitors; INSTIs, integrase strand transfer inhibitors; PIs, protease inhibitors.

## Data Availability

No new data were created or analyzed in this study. Data sharing is not applicable to this article.

## Supplementary Materials

The following supporting information can be downloaded at: https://www.mdpi.com/article/10.3390/jcm15020651/s1, Table S1: Detailed search strategy for electronic databases; Table S2: Excluded articles and reasons for exclusion; Table S3: Quality assessment of included studies; File S1: PRISMA 2020 checklist.

## Abbreviations

The following abbreviations are used in this manuscript:

NSPT	Non-surgical periodontal therapy
HIV	Human immunodeficiency virus
PLWH	People living with HIV
HAART	Highly active antiretroviral therapy
NNRTIs	Non-nucleoside reverse transcriptase inhibitors
PIs	Protease inhibitors
TDF	Tenofovir
3TC	Lamivudine
ATV	Atazanavir
OHI	Oral hygiene instruction
SRP	Scaling and root planing
SPT	Supportive periodontal treatment
PDT	Photodynamic therapy
MG	Malachite green
PPD	Probing pocket depth
CAL	Clinical attachment level
BoP	Bleeding on probing
PI	Plaque index
GBI	Gingival bleeding index
BI	Bleeding index
LF	Lactoferrin
HST	Histatin
GCF	Gingival crevicular fluid
