# Supplementary material for: Non-Surgical Periodontal Treatment Outcomes in Patients with HIV Under Antiretroviral Therapy: A Systematic Review
Source: J Clin Med. 2026 Jan 14;15(2):651. doi: 10.3390/jcm15020651 (PMC12841715; doi:10.3390/jcm15020651)
Supplement: Supplementary file 1 [file jcm-15-00651-s001.zip › jcm-4057692-Supplementary Materials Tables.pdf]

Non-Surgical Periodontal Treatment Outcomes in Patients with HIV Under Antiretroviral Therapy: A Systematic Review

Thaleia Angelopoulou [1] and Yiorgos A. Bobetsis [2,*]

[1] School of Medicine, National and Kapodistrian University of Athens, 11527 Athens, Greece;
thangelop@yahoo.gr
[2] Department of Periodontology, School of Dentistry, National and Kapodistrian
University of Athens, 11527 Athens, Greece
* Correspondence: ybobetsi@dent.uoa.gr; Tel.: +30-6936613292

Supplementary Material

Table S1. Detailed search strategy for electronic databases.

Search strategy and the date it was performed in the chosen databases.

Electronic databases

Database	Search (December 22, 2025)	Studies
**PubMed**	("Periodontitis"[MeSH] OR "Periodontal Diseases"[MeSH] OR "Dental Scaling"[MeSH] OR "Debridement"[MeSH] OR "non-surgical periodontal therapy"[tiab] OR "periodontal therapy"[tiab] OR "periodontal treatment"[tiab] OR "scaling and root planing"[tiab] OR "periodontal disease"[tiab] OR "periodontitis"[tiab] OR SRP[tiab] OR PDT[tiab]) AND ("HIV Infections"[MeSH] OR "HIV-1"[MeSH] OR HIV[tiab] OR "HIV positive"[tiab] OR "HIV infection"[tiab] OR "HIV infected"[tiab]) AND ("Treatment Outcome"[MeSH] OR "treatment outcomes"[tiab] OR "clinical outcomes"[tiab] OR "impact"[tiab] OR "CD4 T-cell counts"[tiab] OR "viral load"[tiab] OR "effect"[tiab] OR "periodontal parameters"[tiab] OR "treatment effect"[tiab] OR "effectiveness"[tiab] OR "post-treatment"[tiab])	260
**Web**	TS=("periodontitis" OR "periodontal disease" OR "non-surgical periodontal therapy" OR "periodontal therapy" OR "periodontal treatment" OR "scaling	250

**of Science**	and root planing" OR "dental scaling" OR "debridement" OR SRP OR PDT) AND TS=(HIV OR "HIV infection" OR "HIV positive" OR "HIV infected" OR "HIV-1") AND TS=("treatment outcomes" OR "clinical outcomes" OR "CD4 T-cell counts" OR "viral load" OR effect OR impact OR "periodontal parameters" OR "treatment effect" OR effectiveness OR "post-treatment")	
**Scopus**	TITLE-ABS-KEY ("periodontitis" OR "periodontal diseases" OR "non-surgical periodontal therapy" OR "periodontal therapy" OR "periodontal treatment" OR "debridement" OR "scaling and root planing" OR srp OR pdt OR "dental scaling") AND TITLE-ABS-KEY (hiv OR "hiv positive" OR "hiv infection" OR "hiv infected") AND TITLE-ABS-KEY ("treatment outcomes" OR "clinical outcomes" OR "impact" OR "effect" OR "CD4 T-cell counts" OR "viral load" OR "effectiveness" OR "periodontal parameters" OR "post-treatment")	495
**Cochrane Library**	(periodontitis OR periodontal disease OR nonsurgical periodontal therapy OR periodontal therapy OR periodontal treatment OR scaling and root planing OR dental scaling OR debridement) AND (HIV OR HIV infection OR HIV positive OR HIV infected OR HIV-1) AND (treatment outcomes OR clinical outcomes OR CD4 OR viral load OR periodontal parameters OR treatment effect OR effectiveness OR post treatment OR impact OR effect)	67

Grey Literature

**BASE**	("periodontitis" OR "periodontal diseases" OR "non-surgical periodontal therapy" OR "periodontal therapy" OR "periodontal treatment" OR "debridement" OR "scaling and root planing" OR SRP OR PDT OR "dental scaling") AND (HIV OR "HIV positive" OR "HIV infection" OR "HIV infected" OR "HIV-1") AND ("treatment outcomes" OR "clinical outcomes" OR "impact" OR "effect" OR "CD4 T-cell counts" OR "viral load" OR "effectiveness" OR "periodontal parameters" OR "treatment effect" OR "post-treatment")	1,011
**ProQuest**	NOFT(("periodontitis" OR "periodontal diseases" OR "non-surgical periodontal therapy" OR "periodontal therapy" OR "periodontal treatment" OR "debridement" OR "scaling and root planing" OR SRP OR PDT OR "dental scaling") AND (HIV OR "HIV positive" OR "HIV infection" OR "HIV infected" OR "HIV-1") AND ("treatment outcomes" OR "clinical outcomes" OR	34

	"impact" OR "effect" OR "CD4 T-cell counts" OR "viral load" OR "effectiveness" OR "periodontal parameters" OR "treatment effect" OR "post-treatment"))	
**GoogleScholar**	("periodontitis" OR "periodontal diseases" OR "non-surgical periodontal therapy" OR "periodontal therapy" OR "periodontal treatment" OR "debridement" OR "scaling and root planing" OR "dental scaling") AND ("HIV" OR "HIV infection" OR "HIV positive" OR "HIV-1") AND ("treatment outcomes" OR "clinical outcomes" OR "CD4 T-cell counts" OR "viral load" OR "periodontal parameters" OR "effectiveness")	100
**Research Gate**	"non-surgical periodontal treatment" "malachite green" "methylene blue" HIV/AIDS	2

Table S2 Excluded articles and reasons for exclusion

Records identified from Electronic Databases	
Full-text articles excluded, with reasons (n=10)	
Author/Year	Reason for exclusion
Gonçalves et al., 2004	1
Gonçalves et al., 2005	1
Noro Filho et al., 2013	1
Noro Filho et al., 2012	1
Valentine et al., 2016	1
Gušić et al., 2016	1
Giovani et al., 2016	3
Shintani et al., 2023	1
Ramos Peña et al., 2022	2
Jordan et al., 2018	1

Reasons for exclusion:

1 - Smoking (n=8)

2 - Data duplication (n=1)

3 - Not-peer reviewed source (n=1)

Records identified from Grey Literature	
Full-text article excluded, with reason (n=1)	
Author/Year	Reason for exclusion
Corassari et al., 2024	1

<u>Reason for exclusion:</u>

1 - Unclear intervention protocol (n=1)

Table S3: Quality assessment of included studies

Outcome and follow-up	Patients (studies), N	Certainty	What happens
Change in Probing Pocket Depth (PPD): Follow-up: up to 15 months	81 (3 non-randomised studies)	⊕◯◯◯ Very low [a,b,d]	Two studies (Jordan 2006; Nobre 2019/2023) found significant within-group PPD reductions after NSPT in HIV^+/HIV^- (Jordan 15 months : HIV^+ 4.2±0.3mm→2.6±0.4mm; HIV^- 4.3±0.3mm→2.6±0.4mm, p=0.0005; Nobre 30/90 days: HIV^+ 2.8±0.6mm→2.2±0.6mm→2.0±0.6mm; HIV^- 2.8±0.6mm→2.6±0.6mm→2.4±0.5mm, p<0.01). Nobre showed a small between-group effect for PPD (p=0.04). Salgado 2017 reported a numerical PPD decrease (4.5±0.5mm→3.9±0.5mm, 1 week) without reported significance.
Clinical Attachment Level (CAL): Follow-up: up to 1,5 months	47 (1 non-randomised study)	⊕◯◯◯ Very low [a,d]	Nobre 2019/2023 reported significant within-group CAL improvement after NSPT (p<0.01). HIV^+: 3.1±0.7mm→2.7±0.7mm→2.6±1.0mm; HIV^-: 3.3±0.9mm→3.1±0.8mm→3.0±0.9mm (30/90 days). No between-group difference (p=0.11).
Bleeding on Probing (BoP): Follow-up: up to 1,5 months	66 (2 non-randomised studies)	⊕◯◯◯ Very low [a,d,e]	Two studies (Jordan 2006; Salgado 2017) assessed BoP following NSPT. Jordan et al. reported BoP reduction in both HIV^+ and HIV^- participants, from >50% bleeding sites at baseline to approximately 20% at 15 months (p < 0.001). Salgado et al. stated that BoP would be evaluated but no numerical data or statistical analysis were reported.
Gingival Bleeding Index (GBI): Follow-up: up to 3 months	66 (2 non-randomised studies)	⊕◯◯◯ Very low [a,d,e]	Two studies (Salgado 2017; Nobre 2019) assessed GBI after NSPT. Salgado et al. showed a significant GBI reduction at 3 months. Nobre et al. reported GBI reduction in both HIV^+ and HIV^- groups in 30 and 90 days, without numerical values being published. Overall, NSPT reduced gingival bleeding within groups.
Bleeding Index (BI): Follow-up: 3 months	47 (1 non-randomised study)	⊕◯◯◯ Very low [a,c,d]	Nobre et al. (2023) reported significant within-group reductions in both HIV^+ and HIV^- participants (p < 0.01). In the HIV^+ group, BI decreased from 35.4 (19.6–58.3)% → 10.3 (3.6–27.8)% → 12.7 (3.9–22.5)% at 30 and 90 days respectively, while in HIV^- participants BI decreased from 29.0 (12.0–44.8)% → 10.7 (6.9–20.7)% → 9.1 (5.0–15.2)%. No significant between-group differences were found. Overall, NSPT significantly improved BI in both groups within the 3-month follow-up.
Plaque Index (PI): Follow-up: up to 15 months	46 (2 non-randomised studies)	⊕◯◯◯ Very low [a,d,e]	Two studies (Jordan 2006; Salgado 2017) evaluated PI following NSPT. Jordan et al. stated that plaque index was measured, however no numerical data or statistical analysis was reported. Salgado et al. reported a numerical PI reduction post-NSPT in both HIV^+ and HIV^- patients, but this change was not statistically significant.

Outcome and follow-up	Patients (studies), N	Certainty	What happens
Viral Load: Follow-up: up to15 months	33 (2 non-randomised studies)	⊕◯◯◯ Very low [a,c,d,e]	Two studies (Jordan 2006; Nobre 2019) evaluated changes in viral load following NSPT. Jordan et al. observed a slight post-treatment reduction over a 15-month follow-up, which was not statistically significant. Nobre et al. reported viral load decrease from 1,444,892.2 ± 423,174.5 to 19,547.4 ± 66,181.4 and 28,380.8 ± 103,229.3 copies/mL at 30 and 90 days, respectively, also without statistical significance ($p = 0.2984$). Both studies indicated stabilization or modest improvement in viral load.
CD4+ lymphocyte count: Follow-up: up to 15 months	33 (2 non-randomised studies)	⊕◯◯◯ Very low [a,c,d,e]	Two studies (Jordan 2006; Nobre 2019) evaluated $CD4^+$ lymphocyte count changes following NSPT. Jordan et al. observed stable $CD4^+$ counts over a 15-month observation period. Nobre et al. reported a statistically significant increase in $CD4^+$ count from 104.7 ± 57.3 to 165.7 ± 110.4 and 195.6 ± 155.2 cells/mm³ at 30 and 90 days post-NSPT ($p = 0.0120$).Both studies suggest a potential short-term immunological benefit or stabilization after NSPT.
CD4/CD8 ratio: Follow-up: 15 months	11 (1 non-randomised study)	⊕◯◯◯ Very low [a,c,d,e]	Jordan et al. (2006) evaluated $CD4^+/CD8^+$ ratio changes following NSPT in HIV^+ patients under HAART. The ratio remained stable throughout the 15-month follow-up, with no statistically significant change. No numerical data were reported.
Salivary lactoferrin (LF): Follow-up: 3 months	47 (1 non-randomised study)	⊕◯◯◯ Very low [a,c,d]	Nobre et al. (2023) measured salivary LF concentrations before and after NSPT in HIV^+ and HIV^- patients. LF decreased slightly over time in both groups without statistical significance (HIV^+ 4.1 (2.6-5.1) µg/mL → 2.6 (1.2-4.3) µg/mL → 2.2 (1.5-4.4) µg/mL; HIV^- 3.0 (1.4-4.7) µg/mL → 2.7 (0.9-5.1) µg/mL → 1.8 (0.9-4.5) µg/mL; Group $p = 0.51$; Time $p = 0.07$).
Salivary histatin (HST): Follow-up: 3 months	47 (1 non-randomised study)	⊕◯◯◯ Very low [a,c,d]	Nobre et al. (2023) measured salivary HST before and after NSPT in HIV^+ and HIV^- patients. In HIV^+ patients, levels changed from 62.6 (34.6-78.8) pg/µL to 59.8 (37.2-84.4) pg/µL and 62.3 (49.7-79.5) pg/µL at 30 and 90 days, respectively. In HIV^- patients, values were lower (22.4 (6.6-32.5) → 20.1 (11.3-33.9) → 20.1 (10.4-31.4) pg/µL). A significant between-group difference was found ($p < 0.01$), without change over time.
Gingival crevicular fluid lactoferrin (GCF LF): Follow-up: 3 months	47 (1 non-randomised study)	⊕◯◯◯ Very low [a,c,d]	Nobre et al. (2023) measured GCF LF following NSPT in HIV^+ and HIV^- patients. In HIV^+ patients, levels changed from 8.8 (5.0-10.7) pg/µL to 10.3 (6.9-13.5) pg/µL and 8.8 (7.3-11.5) pg/µL at 30 and 90 days, respectively. In HIV^- patients, LF levels were lower, measured at 6.7 (4.8-8.4) pg/µL→ 5.4 (4.5-7.7) pg/µL→ 5.7 (5.0-7.5) pg/µL. A significant between-group difference was observed ($p = 0.01$).
Gingival crevicular fluid histatin (GCF HST): Follow-up: 3 months	47 (1 non-randomised study)	⊕◯◯◯ Very low [a,c,d]	Nobre et al. (2023) measured GCF histatin before and after NSPT in HIV^+ and HIV^- patients. In HIV^+ patients, levels changed from 3.7 (1.9-5.7) pg/µL to 3.4 (1.8-5.8) pg/µL and 3.3 (1.7-5.4) pg/µL at 30 and 90 days, respectively. In HIV^- patients, values were slightly higher (4.4 (2.1-6.8) pg/µL→ 4.6 (2.4-6.9) pg/µL → 4.3 (2.5-6.7) pg/µL). No significant between-group difference ($p = 0.12$) or change over time was detected.

Reasons for downgrading: a. risk of bias, b. inconsistency, c. indirectness, d. imprecision, e. publication bias
